# Factors associated with treatment modification during the first year of contemporary HAART

**DOI:** 10.1186/1758-2652-13-S4-P15

**Published:** 2010-11-08

**Authors:** N Martin, I Perez-Valero, B San Jose Valiente, M Mora, JI Bernardino-Serna, J Gonzalez, F Gaya, FX Zamora, JM Pena, JR Arribas

**Affiliations:** 1 Hospital La Paz/Autónoma University School of Medicine. IdiPAZ, Internal Medicine, Madrid, Spain; 2 Hospital La Paz/Autónoma University School of Medicine. IdiPAZ, HIV Unit, Madrid, Spain; 3 Hospital La Paz/Autónoma University School of Medicine. IdiPAZ, Biostatistical Unit, Madrid, Spain; 4 Hospital La Paz/Autónoma University School of Medicine. IdiPAZ, Infectious Diseases Unit, Madrid, Spain; 5 Hospital La Paz/Autónoma University School of Medicine. IdiPAZ, Madrid, Spain

## Purpose of the study

To estimate the short-term probability of HAART change and to evaluate factors associated with treatment modification during the first year of contemporary HAART.

## Methods

We evaluated by logistic regression and Cox proportional model analysis factors associated to treatment modification during the first year of HAART in antiretroviral naïve patients from a single HIV unit in Madrid. Variables included in the analysis were: Basic sociodemographic characteristics, data on the clinical course, VHC coinfection, antiretroviral therapy and immunologic and virologic variables.

## Results

From Jan/06 to Dec/09, 301 patients started HAART (mean age 38.6, 75.4% male). Median CD4: 246, mean viral load: 4.67 logs, 22.3% HCV coinfected. Patients started HAART including TDF/FTC (84.7%), ABC/3TC (8.3%), AZT/3TC (7%), EFV (53.5%), NVP (7.7%), LPV/r (23.6%), DRV/r (2.0%) ATV/r (2.7%), FPV/r (6.3%), SQV/r (0.5) and RAL (3.7%). One-year probability of HAART modification was 0.26 (95%CI 0.21-0.26). Reasons for HAART modification were toxicity (11%), simplification (10.6%, including 8.31% of patients who switched from TDF/FTC+EFV to TDF/FTC/EFV), Lack of efficacy (1.3%) and other (i.e. poor adherence, pregnancy termination 3.3%). Most common toxicities leading to HAART modification were skin rash (3.32%), CNS adverse events (1.66%), gastrointestinal (1.33%) lipoatrophy (1.66%), renal (1%), and osteopenia (1%). Patients who modified HAART were less likely to achieve 1 year viral load suppression than patients who didn't change HAART (83.5% vs 94.6%, p = 0.02) with no differences in CD4 cell recovery. Multivariant logistic regression analysis showed that a prior AIDS-defining condition [OR 1.90 (1.07-3.37)] and AZT/3TC as nucleoside backbone [4.81 (1.88-12.3)] were significantly associated to HAART modification. A sensitivity analysis excluding TDF/FTC+EFV to TDF/FTC/EFV switches showed two additional factors associated to treatment modification: female sex [2.62 (1.30-5.27)] and time since HIV diagnosis.

## Conclusions

Treatment modification is still common during the first year of HAART occurring in one quarter of patients. While lack of efficacy of HAART is an uncommon reason for change, toxicity and treatment simplification remain important reasons for change. Women, patients with a prior AIDS diagnosis and patients receiving AZT/3TC as a backbone were more likely to change therapy.

